# Immersive Virtual Reality for 3D Cephalometric Landmarking Across Low-Dose and Ultra-Low-Dose CBCT: A Pilot Comparison with a Conventional Computer Interface

**DOI:** 10.3390/diagnostics16162493

**Published:** 2026-08-07

**Authors:** Jorma Järnstedt, Helena Mehtonen, Jari Kangas, Hanna Naukkarinen, Kimmo Ronkainen, John Mäkelä, Sakarat Nalampang, Phattaranant Mahasantipiya, Arnon Charuakkra, Wannakamon Panyarak, Irina Rinta-Kiikka, Roope Raisamo

**Affiliations:** 1 Department of Radiology, Tampere University Hospital, Wellbeing Services County of Pirkanmaa, 33250 Tampere, Finland; 2 Faculty of Medicine and Health Technology, Tampere University, 33014 Tampere, Finland; 3 TAUCHI Research Center, Faculty of Information Technology and Communication Sciences, Tampere University, 33100 Tampere, Finland; 4 Faculty of Medicine, University of Helsinki, 00014 Helsinki, Finland; 5 Division of Oral and Maxillofacial Radiology, Department of Oral Biology and Diagnostic Sciences, Faculty of Dentistry, Chiang Mai University, Chiang Mai 50200, Thailand

**Keywords:** cone-beam computed tomography, virtual reality, cephalometric landmarking, observer variability, dose optimisation, deep learning, craniomaxillofacial surgery

## Abstract

**Background:** Three-dimensional cephalometric landmarking provides the spatial reference framework for computer-aided surgical simulation in craniomaxillofacial (CMF) surgery. Conventional workflows rely on two-dimensional computer interfaces (CIs), yet imaging data are inherently volumetric, and repeated CBCT imaging creates pressure to minimise patient radiation exposure. Immersive virtual reality (VR) offers a more intuitive environment for spatial tasks, yet the feasibility of ultra-low-dose (uLD) protocols for cephalometric landmarking in CI and VR remains unevaluated. This pilot study evaluated accuracy, reproducibility and workload across low-dose (LD) and uLD CBCT protocols in both environments. **Methods:** Four CMF radiologists placed ten 3D cephalometric landmarks on 20 CBCT datasets across three rounds. Round 1 used deep learning-predicted coordinates as ground truth. Rounds 2–3 assessed blind reproducibility. Workload was evaluated using the NASA Task Load Index. **Results:** Median Round 1 accuracy was 1.00 mm in both environments; mean CI accuracy was 1.05 mm and mean VR accuracy was 2.03 mm after outlier exclusion. Log analysis identified controller slips and software logging errors as a primary VR outlier source (22/793, 2.77%). Reproducibility was higher in CIs; VR medians remained clinically relevant. Dose level had no meaningful effect. The NASA-TLX showed decreasing VR workload across sessions, with AI-guided landmarking associated with the lowest mental demand. **Conclusions:** VR-based landmarking is feasible: its median accuracy was comparable to that of CIs and radiologists responded positively. Reproducibility was lower in VR, attributable to software constraints rather than the visualisation modality. The uLD protocol performed comparably to LD, supporting dose optimisation. Integration of AI assistance within VR represents the most promising direction for further development.

## 1. Introduction

Three-dimensional (3D) computer-assisted surgical simulation (CASS) has become an essential component of modern craniomaxillofacial (CMF) planning, enabled by advances in volumetric imaging—most notably cone-beam computed tomography (CBCT) [1,2,3]. CASS is applied across orthognathic correction, trauma reconstruction and tumour resection [4,5,6,7,8,9,10,11], improving both procedural efficiency and surgical accuracy [4,12] while reducing the risk of complications such as excessive bleeding or temporary and permanent nerve injury [13,14,15,16,17,18]. Despite this progress, clinical interpretation and virtual planning workflows still rely heavily on two-dimensional (2D) computer interfaces (CIs) operated via mouse and keyboard, even though both the imaging data and the anatomical structures of interest are inherently 3D. This mismatch between volumetric data and 2D-based visualisation places substantial demands on clinicians’ spatial reasoning and can make diagnostic interpretation and CASS procedures time-consuming and prone to error [19].

Cephalometric analysis provides quantitative measurements of skeletal and dental landmarks to characterise facial morphology and guide treatment planning in orthodontics and orthognathic surgery [20,21]. Originally based on 2D lateral cephalograms, it enables assessment of skeletal discrepancies, dental relationships and soft-tissue profiles essential for diagnosing malocclusions and planning corrective interventions [20]. Key angular measurements quantify anteroposterior and vertical jaw relationships relative to the cranial base, forming the basis for skeletal classification and surgical decision-making [22,23]. CBCT has become the preferred imaging modality for 3D CMF CASS, offering substantially lower radiation dose than conventional CT while providing sufficient spatial resolution for osseous landmark identification [24,25,26]. Unlike conventional 2D cephalometry, which projects the entire craniofacial skeleton onto a single plane, 3D analysis allows independent assessment of bilateral structures, direct measurement of asymmetries and volumetric evaluation of the facial skeleton [27,28]. This is particularly relevant in orthognathic surgery, where asymmetric skeletal deformities are common and 2D projections may mask or distort clinically significant differences [29,30]. In addition, 3D CBCT-based CASS enables the fusion of volumetric imaging with digital dental arch models, requiring accurate and reproducible reference landmarks to minimise surgical inaccuracies [31].

Despite its clinical importance, manual cephalometric landmarking is inherently operator-dependent. Even in 2D cephalometry, inter- and intraobserver variability has been well documented, with landmark placement errors influenced by image quality, landmark definition ambiguity and observer experience [32]. In 3D CBCT-based analysis, these challenges are compounded: landmarks must be identified on curved three-dimensional surfaces rather than projected outlines, anatomical variability is more apparent, and the absence of standardised 3D landmark definitions further contributes to interobserver discordance [33,34]. Clinically, landmark placement errors of 2 mm or less are generally considered acceptable for CASS applications, as this threshold corresponds to the precision required for accurate surgical simulation and outcome prediction [6,23].

Recent advances in deep learning (DL) have enabled automated 3D cephalometric landmarking from CBCT scans with accuracy approaching or meeting clinical thresholds, including validation across multi-ethnic, multi-centre and multi-device datasets [35]. More recently, a mean radial error consistently below 1.3–1.4 mm has been reported across multi-centre spiral CT and CBCT cohorts, including complex cases involving malocclusion or metal artefacts [36].

However, fully automated landmark placement cannot yet replace expert human judgement in clinical workflows: automated predictions require manual review, correction of systematic errors and adjustment for anatomical variants or pathological changes [35]. Models shown to measurably improve specialist landmarking speed and proficiency still require further prospective clinical validation before routine use, as their authors acknowledge [36]. The reproducibility and accuracy of manual 3D cephalometric landmarking therefore remain clinically relevant, particularly when evaluated under novel visualisation conditions such as immersive virtual reality (VR).

Beyond CMF surgery, VR has been explored across medical specialties as a tool for enhancing spatial understanding and procedural planning. In neurosurgery, VR has improved preoperative comprehension of tumour and vascular anatomy [37,38]; in orthopaedics, it has facilitated fracture analysis and implant planning for complex 3D geometry [39]; and in the dental domain, immersive 3D visualisation has enhanced spatial understanding of implant positioning [40]. A consistent finding across specialties is that VR confers advantages in tasks requiring three-dimensional spatial reasoning—precisely the conditions present in CMF cephalometric landmarking [41,42]. However, a recent scoping review of clinical VR and AR applications in radiology found that the evidence base across these specialties remains largely at a feasibility stage, characterised by small sample sizes and inconsistent reporting quality [43]—underscoring the need for the kind of detailed, transparent accuracy and reproducibility reporting undertaken in the present pilot study.

VR has been particularly well established as an educational tool in undergraduate dental and anatomical training, where immersive visualisation has been shown to improve conceptual understanding and retention compared with conventional interfaces [44]. This is consistent with findings from a recent related work using the same research platform and context [45]. The present study addresses a distinct question—diagnostic accuracy and reproducibility in a clinical rather than an educational context—though the underlying spatial-reasoning demands are closely related, and the interaction and visualisation challenges identified here are likely equally relevant to postgraduate specialist training in orthodontics, oral and maxillofacial surgery and radiology.

Within the CMF field, VR remains an area of active but limited research. Existing studies have primarily addressed feasibility, usability or interaction methods rather than providing validated diagnostic or planning performance data [21,46,47]. Only a small number of studies have specifically examined VR in orthognathic surgical contexts [21,46], and most VR platforms used in medical research lack DICOM calibration—raising questions about the diagnostic reliability of images viewed through commercial headsets [29,41,42]. Technical image quality indicators and optimal radiation dose parameters for STL model generation in CMF visual surgical planning have been established in a dedicated phantom study [48]. A subsequent pilot study confirmed that a dedicated low-dose (LD) CBCT protocol provided diagnostically acceptable image quality in both CI and VR environments, with observer agreement and reliability comparable to DICOM-calibrated monitors [49]. These findings established a prerequisite for the present study: that radiological image quality in a commercial VR headset is sufficient for CMF cephalometric tasks—and that dose can be further reduced to ultra-low-dose (uLD) levels without compromising diagnostic utility.

Interaction methods represent a critical prerequisite for clinical VR adoption in CMF tasks. A systematic evaluation of four interaction modalities demonstrated that the VR controller achieved the best combination of accuracy (1.73 mm), task completion time and user preference among CMF radiologists, while hand tracking remained insufficient for clinical use (mean error: 4.52 mm) [50]. The NASA Task Load Index (NASA-TLX) assessments from the same research programme further indicated that perceived workload decreased progressively over successive VR sessions, suggesting genuine adaptation to the interface [50]. Critically, the feasibility of uLD CBCT protocols for VR-based cephalometric assessment remained unevaluated—a gap relevant across all patient groups, as the as low as reasonably achievable (ALARA) principle mandates continuous dose optimisation in any imaging workflow [25,26].

Taken together, these findings establish the technical prerequisites for VR-based CMF cephalometric analysis, i.e., a validated interaction modality [50], acceptable image quality across dose levels [49] and an automated DL reference standard for landmark accuracy assessment [35], which has been previously validated as a reliable comparator for manual landmark placement. However, a critical gap remains: the accuracy and reproducibility of manual 3D cephalometric landmarking in an immersive VR environment have not been examined. It is unknown whether clinicians achieve accuracy and reproducibility comparable to conventional CI-based cephalometry when operating in VR, nor whether the known challenges of 3D cephalometric landmarking are exacerbated or mitigated by the immersive 3D environment. Furthermore, the feasibility of uLD protocols for cephalometric tasks in both CI and VR remains unexplored.

To our knowledge, this is the first study to directly compare manual 3D cephalometric landmarking accuracy and reproducibility between CI and VR under dose-stratified, blinded, repeated-measures conditions, using a validated VR interaction modality and a DL-derived reference standard [35,50].

A pilot study design was considered appropriate to establish feasibility, characterise effect sizes and inform the design of future larger-scale investigations. The aim of this pilot study was to evaluate the accuracy and reproducibility of CBCT-based 3D cephalometric landmarking across two evaluating environments—conventional CI and immersive VR environments—and across two CBCT dose levels—low-dose (LD) and ultra-low-dose (uLD). Accuracy was assessed by comparing radiologists’ landmark placement with DL-predicted reference coordinates, enabling analysis of both environment-related and observer-related differences. Reproducibility was examined through two blinded landmarking rounds to determine intra- and interobserver variation in each environment and dose level. In addition, user experience and perceived workload were collected using qualitative feedback and the NASA-TLX [51] to evaluate the practical feasibility of VR-based 3D cephalometry for future orthognathic surgical planning and orthodontic assessment.

## 2. Materials and Methods

### 2.1. Patient Data

This retrospective study utilised previously acquired clinical CBCT datasets; no patients were imaged specifically for research purposes. Ethical approval requirements and data-usage permissions are detailed in the Institutional Review Board Statement. All CBCT scans were retrieved from the Picture Archiving and Communication System (PACS) of Tampere University Hospital, Finland, and all patient data were pseudonymised prior to analysis.

The dataset consisted of 20 full-facial CBCT scans, i.e., 10 acquired for orthognathic surgical planning and 10 for orthodontic assessment and monitoring, reflecting the two clinical contexts in which the evaluated dose protocols are routinely applied. The LD protocol corresponds to the standard dose level used at Tampere University Hospital for orthognathic planning, as established in a previous evaluation of CBCT image quality for CMF CASS [44], and was therefore used as the primary reference. The uLD protocol represents a dose-optimised alternative primarily employed for orthodontic assessment, where repeated CBCT imaging is common, and radiation minimisation is a particular priority.

### 2.2. CBCT Imaging Protocols

All scans were acquired using a Planmeca Viso G7 CBCT system (Planmeca Oy, Helsinki, Finland) with a voxel size of 300 μm. Two imaging protocols were used. The LD protocol used 90 kVp, 14 mA and 4.5 s exposure. The uLD protocol used 120 kVp, 3–5 mA and 2.5–3.2 s, a vendor-defined dose-optimised protocol. Radiation dose was quantified using the dose–area product (DAP), reflecting both the delivered X-ray energy and the irradiated field size. The field of view varied according to clinical indication and patient size. Summary exposure parameters and mean DAP values are presented in Table 1.

### 2.3. Participants

Four board-certified CMF radiologists (E1–E4) with 16–40 years of clinical experience participated in all landmarking rounds. Participant selection followed the following criteria:

• **Inclusion:** Board certification in CMF radiology; extensive experience in CBCT interpretation and cephalometric analysis; familiarity with both conventional 3D imaging workflows and VR-based medical imaging environments; availability to complete the full multi-round protocol across both evaluating environments and both CBCT dose levels.
• **Exclusion:** None applied beyond the inclusion criteria above; given the limited pool of radiologists meeting these qualifications within the two participating institutions (Section 5, Institutional Review Board Statement), no further exclusion was possible or considered appropriate.

Each radiologist completed all landmarking tasks independently across both evaluating environments (CIs and VR) and both CBCT dose levels (LD and uLD). Sample size was determined pragmatically by the availability of board-certified CMF radiologists able to complete the full multi-round protocol, rather than by a priori power calculation, consistent with the pilot, feasibility-focused aim of this study.

### 2.4. Study Design

**Figure 1:**
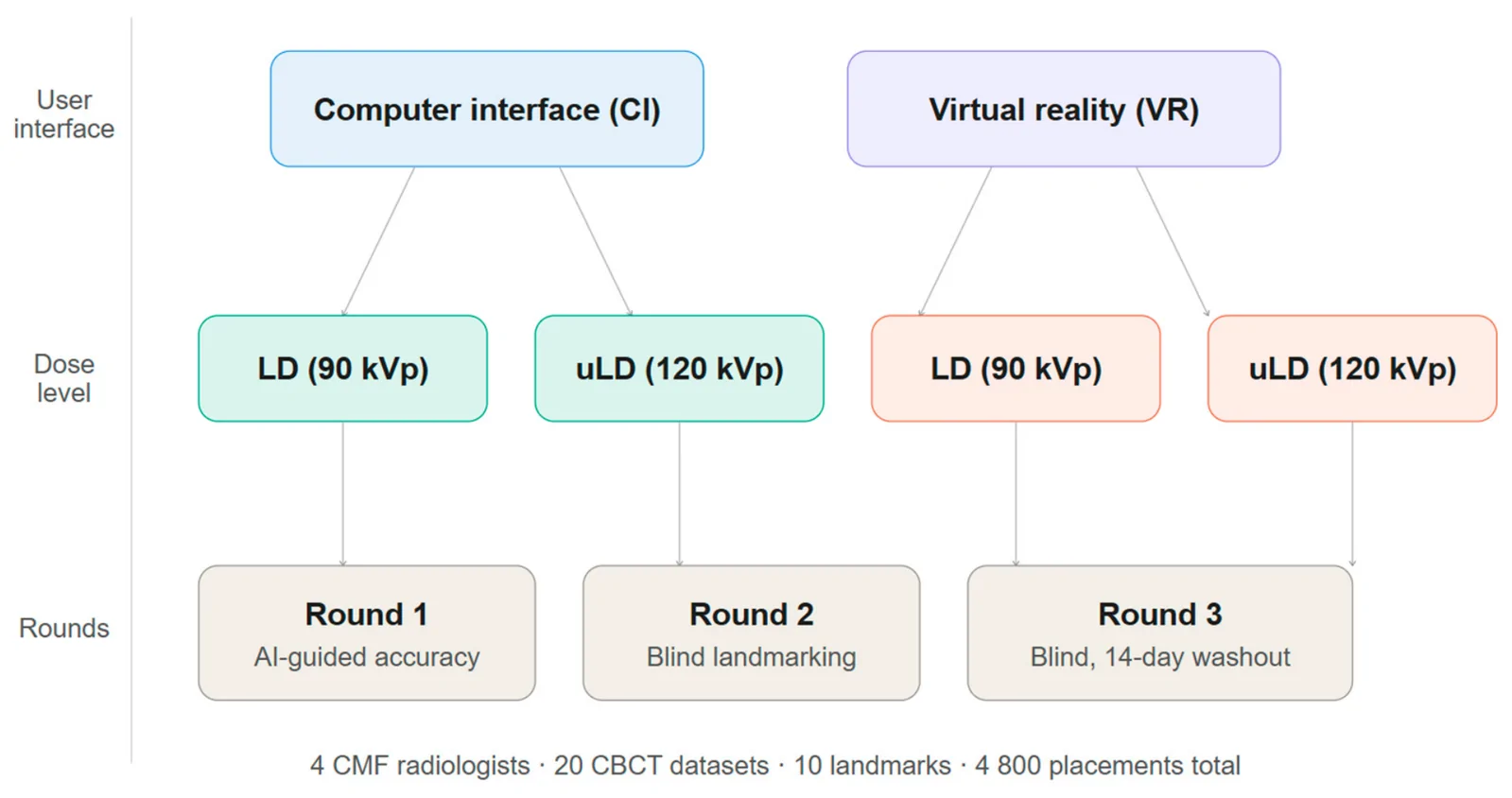
Experimental workflow comparing computer interface (CI) and virtual reality (VR) across dose levels and rounds of testing. R1 = Round 1 (AI-guided accuracy assessment); R2 = Round 2 (blind landmarking, interobserver reproducibility); R3 = Round 3 (blind landmarking, 14-day washout; intra- and interobserver reproducibility).

Each radiologist completed 3D cephalometric landmarking in both evaluating environments and across both CBCT dose levels in three sequential rounds. Round 1 assessed accuracy relative to a DL reference; Round 2 assessed unaided anatomical landmarking without reference points; and Round 3 repeated the blind protocol after a washout period of at least 14 days. Intraobserver reproducibility was derived from comparisons between Rounds 2 and 3; interobserver reproducibility was assessed within each blind round. To minimise recall and learning effects, each radiologist was presented with a unique randomised ordering of all 20 skull datasets in every round. The CI/VR and LD/uLD conditions were independently counterbalanced across participants using a balanced Latin square design, ensuring that no systematic order effects confounded the comparisons. The evaluation process is shown in Figure 1.

### 2.5. Cephalometric Landmarks

Ten standard 3D cephalometric landmarks were used across all rounds and environments: Nasion (N), Condylion Right (CoR), Condylion Left (CoL), Posterior Nasal Spine (PNS), Anterior Nasal Spine (ANS), Subspinale (A-point), Gnathion (Gn), Menton (Me), Gonion Right (GoR) and Gonion Left (GoL). These ten landmarks were selected to provide comprehensive coverage of the craniofacial skeleton across the cranial base, maxilla and mandible, and represent standard osseous reference points used in CMF surgical planning and orthodontic assessment [27,28].

In both environments, the landmarks were presented in an environment-specific structured tray with identical nomenclature.

### 2.6. DL Reference Landmarks

Ground-truth reference coordinates for Round 1 were obtained from a previously validated DL model for automated 3D cephalometric landmarking from CBCT scans [35]. An automated DL reference was preferred over expert consensus as ground truth to avoid circular comparison: using the same observers’ consensus as both reference and test measurement would conflate the reference standard with the quantity being measured.

DL coordinates for all ten landmarks were exported directly and displayed as small reference spheres visible only during Round 1. No manual adjustments were made to the DL coordinates. In Rounds 2 and 3, reference points were hidden to allow unbiased anatomical landmarking.

### 2.7. Evaluating Environments

All cephalometric landmarking tasks were performed in two distinct evaluation environments: a CI environment and an immersive VR environment. Both environments utilised identical volumetric CBCT datasets and 3D skull models to ensure full methodological comparability.

**Figure 2:**
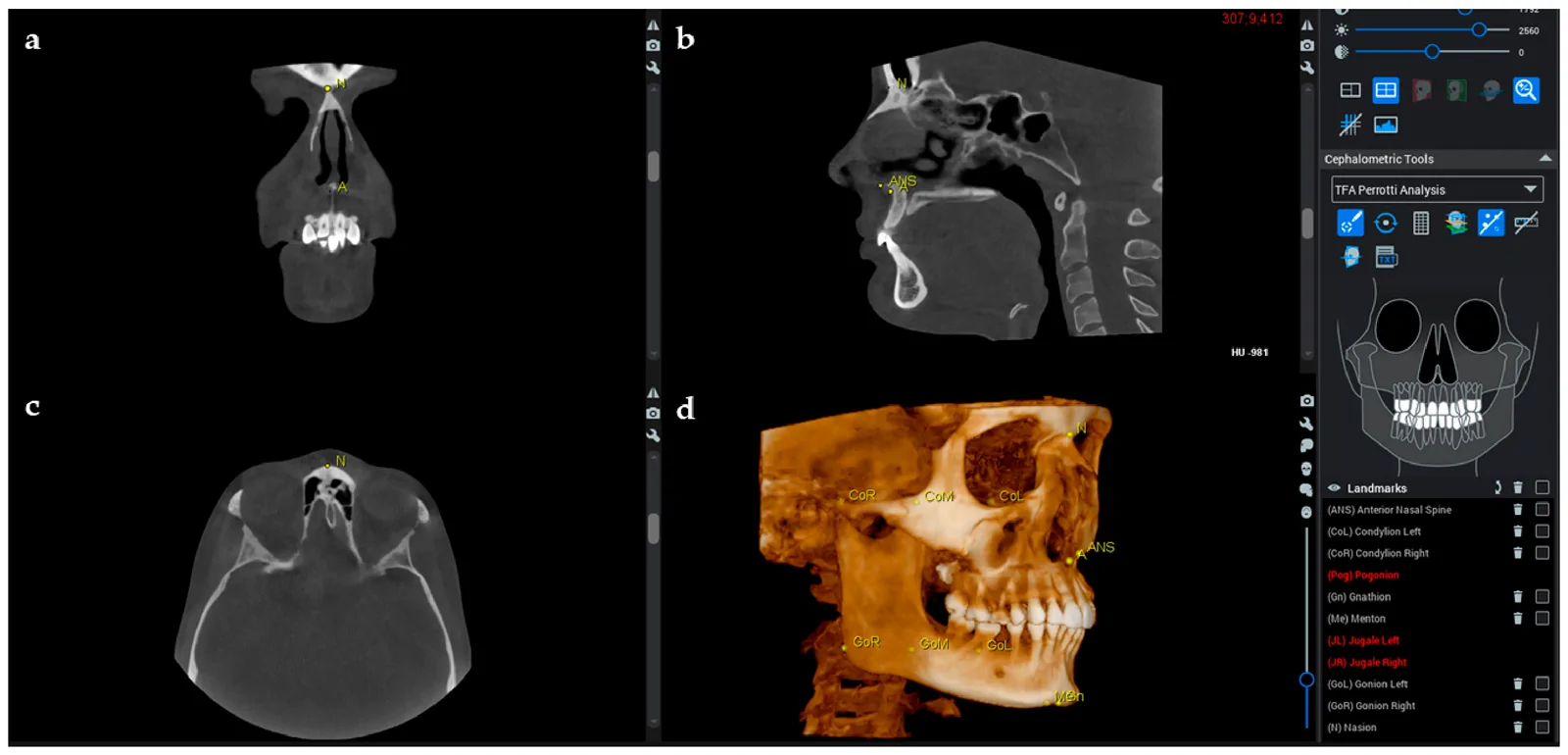
The CI environment: Romexis 3D Ceph showing (**a**) coronal, (**b**) sagittal and (**c**) axial MPR planes at the level of N, A-point and ANS, and (**d**) the 3D surface-rendered skull model, with a structured landmark tray on the right displaying the cephalometric landmarks available for placement. Imaging protocol: uLD (120 kVp, 5 mA, 3.2 s).

The CI environment was implemented using Planmeca Romexis^®^ 3D imaging software (version 6.3.0.2; Planmeca Oy, Helsinki, Finland) on a Barco MDRC-2224 BL 24-inch DICOM-calibrated monitor (1920 × 1200 resolution; Barco NV, Kortrijk, Belgium). Radiologists manipulated the skull in the dedicated Romexis 3D Ceph (cephalometric) mode, which provided a freely rotatable 3D skull model together with fixed multiplanar reconstruction (MPR) views in axial, coronal, and sagittal planes. Standard image rendering and contrast adjustment tools were also available throughout the analysis. All anatomical landmarks were presented in a structured landmark tray in a predefined order, and native Romexis point-placement tools were used throughout. The CI environment and landmark tray are shown in Figure 2.

**Figure 3:**
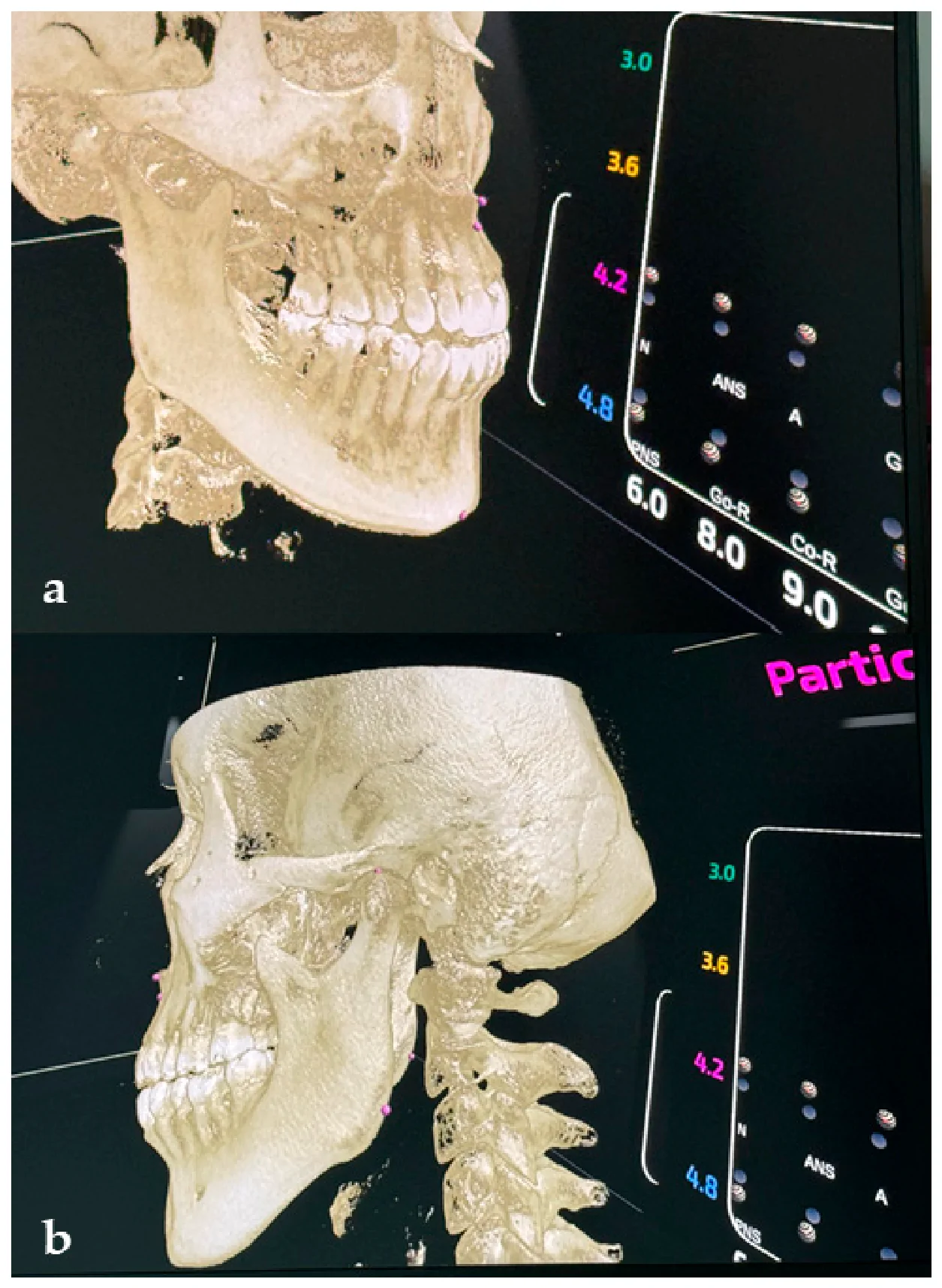
The VR environment: Romexis VR showing (**a**) an oblique lateral view with A-point, ANS and Gn visible on the surface-rendered skull model, and (**b**) a posterior view showing GoL and CoL. A structured landmark tray on the right displays the cephalometric landmarks available for placement. Imaging protocol: LD (90 kVp, 14 mA, 4.5 s).

The VR environment was implemented using a modified version of the Planmeca VR Romexis^®^ platform, originally developed for dental implantology [39], and built on the Unity 3D development platform (version 2021.1; Unity Technologies, San Francisco, CA, USA). The VR module is not independently versioned from the parent Romexis release. Landmarking was performed using a Meta Quest 3 headset (Meta Platforms, Menlo Park, CA, USA; per-eye resolution 2064 × 2208 pixels) with Meta Touch Plus controllers, running the manufacturer’s standard firmware with no custom modifications, which enabled natural hand-guided 3D point placement directly within the immersive skull volume, but the landmarking was only possible on the 3D model. The VR interface included a dedicated landmark tray mirroring the CI structure, ensuring identical nomenclature across environments. The system was run on an MSI Raider GE78HX workstation (Micro-Star International, New Taipei City, Taiwan) equipped with an Intel i9 processor, 32 GB RAM and an NVIDIA RTX 4080 GPU (NVIDIA Corporation, Santa Clara, CA, USA). The VR environment and landmark tray are shown in Figure 3.

### 2.8. Data Collection Protocol

In Round 1, radiologists placed landmarks as accurately as possible with the DL reference points visible. These placements were used to evaluate accuracy relative to the DL reference across environments (CIs vs. VR), dose levels (LD vs. uLD) and radiologists.

In Round 2, DL reference points were hidden, and radiologists landmarked skulls based on anatomical knowledge alone. Comparisons between Rounds 1 and 2 quantified the effect of AI guidance on placement accuracy in each environment.

Round 3 repeated the blind landmarking protocol of Round 2 after a washout period of at least 14 days, ensuring that intraobserver comparisons reflected genuine reproducibility rather than short-term memory of previous placements. Intraobserver reproducibility was evaluated by comparing each radiologist’s landmark coordinates between Rounds 2 and 3 under identical blind conditions. Interobserver reproducibility was assessed from pairwise 3D Euclidean distances between all radiologists’ coordinates for each landmark, round and condition, providing a comprehensive measure of agreement across both blind rounds.

### 2.9. Statistical Analysis

All analyses are descriptive; the small number of patients and expert evaluators precluded inferential statistical testing, and no *p*-values or confidence intervals are reported, as the sample size (four evaluators, twenty cases) was not determined by, nor sufficient for, formal precision estimation (see also Section 2.3). CI landmark coordinates were recorded directly in millimetres by the CBCT software (Planmeca Romexis^®^ 3D imaging software (version 6.3.0.2)). VR landmark coordinates were recorded in two parallel coordinate systems: a global system relative to the virtual room space (WorldSpace), used as the primary data source, and a local system relative to the skull model itself (LocalSpace). All accuracy and reproducibility analyses were based on WorldSpace coordinates, converted from Unity units to millimetres by multiplication by 1000. The VR skull model is rendered directly from the same underlying CBCT volume used in the CI environment, using a fixed, deterministic coordinate mapping rather than a separately fitted rigid registration between independently acquired datasets; no residual registration error in the conventional image-to-image alignment sense therefore arises. Where WorldSpace entries were absent due to software logging failures, LocalSpace entries confirmed that the placements had been performed; such cases were excluded from coordinate-based analyses and reported as logging failures rather than evaluator omissions. All 3D Euclidean distances were calculated in millimetres after conversion, and all coordinates were exported as standardised (x, y, z) triplets. Continuous outcomes are summarised as means, medians and ranges, with the median as the primary descriptor due to outlier influence, particularly in VR. Landmark placements exceeding 10 mm from the reference standard were defined as outliers, consistent with the clinically accepted accuracy threshold for CASS applications.

### 2.10. NASA-TLX Workload Assessment

**Figure 4:**
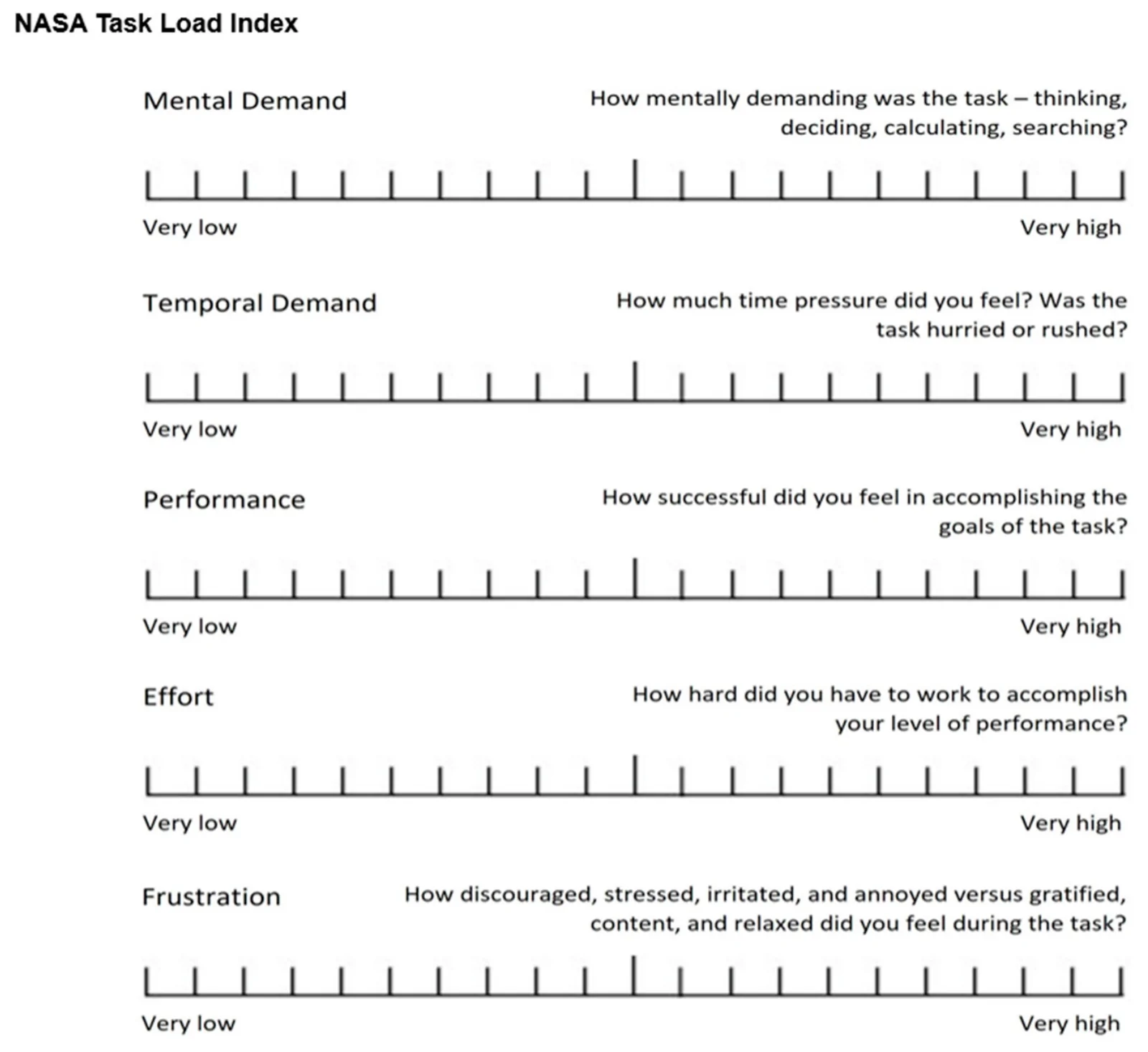
The NASA-TLX questionnaire applied in the present study, covering five subscales (mental demand, temporal demand, performance, effort, frustration) rated from 0 (very low) to 20 (very high).

After each of the three landmarking sessions in both CIs and VR, radiologists completed the modified NASA-TLX [47], as previously described [46], rating mental demand, temporal demand, perceived performance, effort and frustration on a scale from 0 (very low) to 20 (very high). This enabled evaluation of subjective workload differences between CIs and VR and assessment of session-to-session changes across the three rounds. Total workload was calculated as the unweighted mean of the five subscale scores (Raw TLX approach), without the original pairwise-comparison weighting procedure. The NASA-TLX scores were summarised descriptively per session and environment. The NASA-TLX instrument is illustrated in Figure 4.

### 2.11. Follow-Up Interviews

Semi-structured interviews were conducted after each VR session to explore usability, workflow differences compared with CI, ergonomic factors, and challenges and advantages of the VR interface. Qualitative interview data were analysed thematically to identify recurring patterns across observers and sessions.

## 3. Results

Four CMF radiologists (E1–E4) participated in all landmarking rounds across both evaluating environments and both CBCT dose levels, yielding a theoretical maximum of 4800 landmark placements. Of these, 4596 (95.8%) were available for analysis: 1556/1600 (97.3%) in Round 1, 1540/1600 (96.2%) in Round 2, and 1500/1600 (93.8%) in Round 3. Placement losses were attributable to software logging and file export issues and are not attributable to evaluator omission. Both mean and median are reported throughout; given the influence of outlier placements on mean values in VR, the median is used as the primary descriptor for VR data. As this is a pilot-scale dataset (four evaluators, twenty cases), all summary statistics are descriptive; no inferential testing, confidence intervals, or reliability coefficients are reported, and reported values should be interpreted as indicative of feasibility rather than as precise or generalisable estimates.

### 3.1. Round 1: Accuracy Relative to the DL Reference

Observer-level Round 1 mean accuracy ranged from 0.56 mm (E2) to 1.26 mm (E4) in CIs and from 1.09 mm (E1) to 2.97 mm (E3) in VR (Table 2, Part A). In CIs, all four observers achieved means below 1.3 mm. In VR, observer-level means were more variable, with E2 and E3 showing the largest distances (2.44 mm and 2.97 mm, respectively); medians were substantially lower for E2 and E3 (0.80 mm and 0.78 mm), reflecting the influence of outlier placements on VR means.

At the landmark level, CI performance was consistent: all ten landmarks achieved a mean distance of ≤2 mm, with Gn and A-point being the most accurate (both 0.97 mm) and GoL being the least accurate (1.16 mm). In VR, median distances were below 1.5 mm for all ten landmarks, while means were the highest for ANS (4.70 mm), CoL (4.01 mm) and CoR (3.14 mm); see Table 2, Part B.

### 3.2. Effect of DL Guidance on Landmark Placement

With DL reference points visible in Round 1, overall mean accuracy was 1.05 mm in CIs and 2.03 mm in VR. Removing the reference in Round 2 produced a marked increase in both environments: CI mean rose to 2.35 mm (median: 2.03 mm) and VR mean to 6.37 mm (median: 3.58 mm). In Round 3, CIs showed a further slight increase (mean: 2.62 mm; median: 2.00 mm), while VR improved modestly (mean: 5.85 mm; median: 3.68 mm), suggesting progressive interface familiarisation. PNS showed a disproportionately elevated VR mean in both blind rounds (15.60 mm and 7.72 mm, respectively), consistent with the outlier pattern observed in intraobserver and interobserver analyses; see Table 3.

### 3.3. Outlier Analysis

Of the possible 800 CI placements in Round 1 (4 evaluators × 20 patients × 10 landmarks), 763 were available for analysis (95.4%). Thirty-seven placements were absent due to software logging and file export issues and are not attributable to evaluator omission. Of the 763 completed CI placements, one (0.13%) exceeded the 10 mm outlier threshold: an isolated Gn misplacement of 19.75 mm in one evaluator. All DL reference coordinates were present for all 800 landmark–patient combinations.

Of the possible 800 VR placements in Round 1, 793 were available for analysis (99.1%). Seven placements were absent from the WorldSpace log entries; in each case, log inspection confirmed the corresponding LocalSpace entry was present, indicating software logging failure rather than evaluator omission. Of the 793 completed VR placements, 22 (2.77%) exceeded the 10 mm outlier threshold, comprising two mechanistically distinct types. Four placements across two evaluators showed extreme coordinate displacements (138–300 mm) at Condylion and Gonion landmarks. In this sample, the mean intercondylar width was 96.3 mm (range: 85–104 mm) and the mean mandibular angle width was 88.5 mm (range: 80–104 mm), derived from CI gold-standard coordinates. The observed displacements therefore exceed the maximum bilateral craniofacial dimensions of any patient in the study, and are consistent with accidental controller input rather than anatomical misidentification.

A further 18 placements in one session showed a systematic superior displacement of ANS (mean: 29.3 ± 1.1 mm; range: 28.2–31.7 mm). The observed offset of ~29 mm exceeded the ANS–A-point distance and did not correspond to any alternative landmark position. The displacement was absent in the first patient (2.1 mm) and from all other landmarks within the same session, consistent with a session-level software logging error.

These two outlier types received different treatments in subsequent analyses: the controller-slip placements represent genuine, if erroneous, user input and were retained throughout, whereas the ANS displacement reflects invalid logged data and was excluded from accuracy analysis. Round 1 outlier sources are fully characterised in Supplementary Table S1.

In Rounds 2 and 3, CI placement completeness was 740/800 (92.5%) and 700/800 (87.5%), respectively, with losses attributable to the same software logging and file export issues and not attributable to evaluator omission. VR placement completeness was 800/800 (100%) in both rounds. Outlier frequencies by condition are summarised in Table 4, and landmark placements deviating >10 mm between Rounds 2 and 3 are summarised per evaluator in Supplementary Table S2, providing observer-level detail for reproducibility analyses.

### 3.4. Intraobserver Reproducibility (Round 2 vs. Round 3)

Observer-level intraobserver mean distances ranged from 1.98 mm (E1) to 3.36 mm (E2) in CIs and from 2.83 mm (E2) to 7.04 mm (E4) in VR (Table 5, Part A). CI intraobserver performance was consistent across observers, with all four achieving means below 3.4 mm. In VR, observer-level means were higher and more variable, with E3 and E4 showing the largest distances (6.29 mm and 7.04 mm, respectively); medians were more stable (2.96 mm for both), indicating the influence of outlier placements on VR means.

At the landmark level, CI intraobserver mean distances ranged from 1.61 mm (PNS) to 4.55 mm (GoL), with a grand mean of 2.63 mm and an overall median of 2.00 mm (Table 5, Part B). In VR, the grand mean was 5.01 mm, and the overall median was 2.66 mm. PNS showed a disproportionately elevated VR intraobserver mean of 19.37 mm (median: 2.50 mm), attributable to outlier placements rather than a systematic error; excluding PNS, means ranged from 2.38 mm (ANS) to 5.49 mm (GoL). In CIs, PNS and ANS showed the lowest landmark-level variability, while GoL and CoR were the least reproducible.

### 3.5. Interobserver Reproducibility (Rounds 2 and 3)

Observer-pair mean distances in CIs ranged from 2.30 mm (E3–E4) to 2.99 mm (E2–E3) in Round 2 and from 3.17 mm (E1–E3) to 3.57 mm (E1–E4) in Round 3 (Table 6, Part A). In VR, observer-pair means were substantially higher, ranging from 5.69 mm (E2–E4) to 9.35 mm (E3–E4) in Round 2 and from 5.18 mm (E1–E2) to 7.81 mm (E3–E4) in Round 3. In both environments, medians were markedly lower than means, reflecting the influence of outlier placements on pair-level distances.

At the landmark level, CI mean interobserver distances ranged from 1.74 mm (ANS) to 3.45 mm (Gn) in Round 2 (grand mean: 2.69 mm) and 1.87 mm (ANS) to 6.34 mm (CoR) in Round 3 (grand mean: 3.39 mm). In VR, means were higher in both rounds (Round 2: 3.15 mm (ANS) to 28.67 mm (PNS), grand mean = 7.51 mm; Round 3: 2.74 mm (ANS) to 12.95 mm (PNS), grand mean = 6.42 mm), with grand medians of 4.16 mm and 4.20 mm, respectively (Table 6, Part B). Landmark-level patterns mirrored intraobserver findings: ANS showed the lowest variation in both environments, while Gn and GoL were the least reproducible in CIs. In VR, Gn showed the highest variability, followed by Me, when PNS is excluded (see Table 6 footnote).

### 3.6. Dose-Level Effects (LD vs. uLD)

Accuracy and reproducibility were comparable between dose protocols in both environments, within the descriptive precision afforded by this pilot sample. In CIs, Round 1 mean accuracy was 1.05 mm for uLD and 1.04 mm for LD, with identical overall medians of 1.00 mm. Intraobserver reproducibility was consistent across dose levels, with overall medians of 1.73 mm (uLD) and 2.00 mm (LD) in CIs and 2.78 mm (uLD) and 2.56 mm (LD) in VR. Dose-stratified results are presented in Table 7.

### 3.7. NASA-TLX Workload Assessment

Across sessions, CI and VR workload followed opposite trajectories. In CIs, total workload increased from Session 1 (AI-guided) to Session 2 (blind landmarking) and recovered partially in Session 3. In VR, total workload decreased progressively across all three sessions, reaching its lowest level in Session 3. Perceived performance was the highest-rated dimension in VR across all three sessions.

In Session 1, total mean workload was comparable between environments. Mental demand was lower in VR than in CIs.

In Session 2, CI workload increased substantially while VR workload remained more moderate. By Session 3, VR frustration had decreased or remained stable for three of four observers while CI mental demand remained elevated across all three sessions. Substantial individual variation was observed throughout.

**Figure 5:**
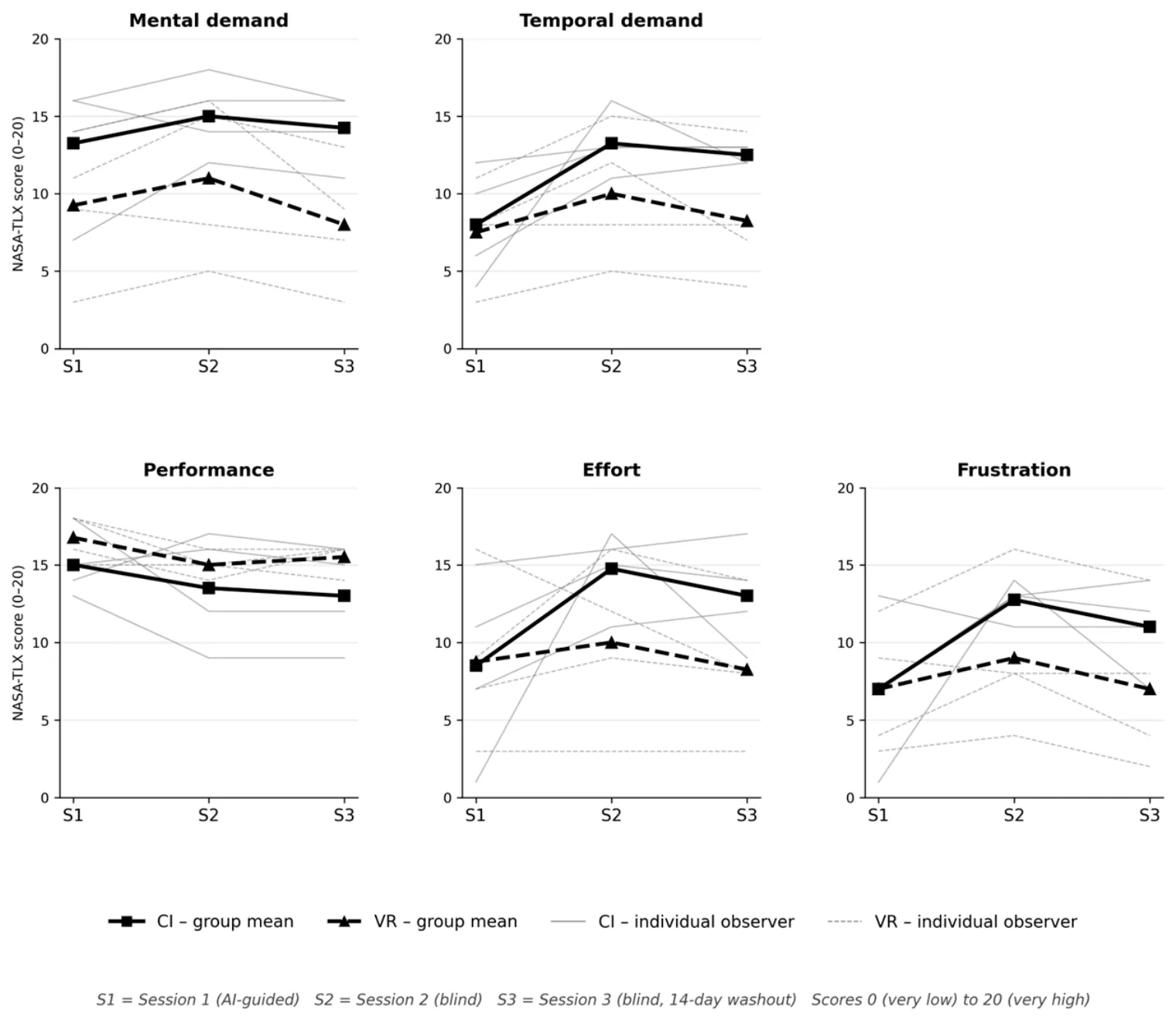
The NASA-TLX subscale scores across three sessions for the computer interface (CI) and virtual reality (VR) environments. Group means: CI (bold solid line, squares); VR (bold dashed line, triangles). Individual observer trajectories shown as thin lines. S1 = Session 1 (AI-guided); S2 = Session 2 (blind); S3 = Session 3 (blind, 14-day washout). Scores 0 (very low) to 20 (very high).

Mean session duration was approximately 60 min for CIs and 45 min for VR. As session duration was not a pre-specified outcome measure of this study, this descriptive observation is reported only at a general level and was not formally tested; it is noted here as directionally consistent with the qualitative reports from multiple observers of a subjective speed advantage in VR. The NASA-TLX subscale trajectories are shown in Figure 5.

### 3.8. Follow-Up Interview Themes

Qualitative feedback identified consistent themes across observers and sessions. In the CI environment, the AI-guided round was praised for its efficiency and landmark guidance. Users valued the ability to cross-verify placements across three CBCT planes simultaneously, which was cited as a key advantage for accuracy and confidence. The primary criticisms across sessions were the time required for manual placement and the number of interaction steps involved. Navigational inflexibility—the inability to adjust the fixed axial, coronal and sagittal planes freely—was specifically identified as a limitation, with users noting that the CI workflow became markedly more demanding and repetitive without AI guidance. The inability to correct individual placements without redoing all markings was also consistently noted.

In the VR environment, the most consistently valued feature was the freedom to rotate and manipulate the 3D skull model freely in all directions. The speed of VR interaction was highlighted as an advantage over CIs by multiple observers, with one observer describing VR as a “fast and furious method” and another noting it was “not a boring method.” Image quality in VR was praised by one observer. By Session 3, multiple observers noted that increasing familiarity with the software reduced task completion time. Physical discomfort was reported by two observers, including eye fatigue, dizziness and occasional nausea, particularly during longer sessions. These symptoms diminished across sessions for most observers.

Specific technical limitations identified consistently in VR included the absence of cross-sectional CBCT slice views and VR landmark spheres perceived as too large. Two distinct controller-related limitations were reported: the imprecision of handheld controller input compared with mouse-based placement, making fine positional adjustments challenging, and the inadvertent displacement of previously placed landmarks when the controller approached adjacent markers during subsequent placements.

## 4. Discussion

Observer accuracy and reproducibility in CIs were consistent with the clinically accepted 2 mm threshold for CASS applications and aligned with published benchmarks for manual and semi-automated 3D cephalometric landmarking [6,22,32,33], providing a reliable baseline against which VR performance can be interpreted.

Observer accuracy in VR, as reflected by median values, was clinically acceptable and approached CI performance across the majority of landmarks. This should be read as a pilot-scale observation rather than a confirmed reliability estimate; the sample size does not support formal precision quantification (e.g., confidence intervals or intraclass correlation coefficients), and larger studies are needed to establish this finding with statistical certainty. Mean values were substantially inflated by a small number of outlier placements concentrated at specific anatomically demanding sites. In VR, CoR, CoL, GoR and GoL—landmarks located at the condylar heads and mandibular angles [52]—were susceptible to unintentional displacement during controller interaction: isolated extreme coordinate displacements of 138–300 mm were directly observed by the supervising researcher during data collection and confirmed by log analysis. These displacements exceeded the maximum craniofacial dimensions observed in this sample, consistent with accidental controller input rather than anatomical misidentification. A second, technically distinct outlier source was a systematic superior displacement of ANS in one session, affecting 18 of 19 patients with high consistency in direction and magnitude; this was attributable to a session-level software logging error rather than observer placement. Both outlier types are identifiable through post-placement review and addressable through targeted interface development, and their influence is reflected in the elevated means observed in subsequent reproducibility analyses.

PNS presented a third outlier source, distinct in mechanism. Defined as the posterior tip of the hard palate [52], it is situated deep within the palatal region and is not always reliably visible on the surface-rendered 3D model used for VR landmarking. In CIs, the landmark can be directly identified on sectional slices using MPR, which resolves depth ambiguity; in the current VR implementation, where landmarking was restricted to the 3D surface model, this cross-sectional reference was unavailable. The elevated PNS outlier rate in VR therefore reflects a rendering constraint specific to surface-based volumetric visualisation rather than observer inconsistency.

The transition from DL-guided to blind landmarking provides a direct measure of AI benefit. In CIs, removing the reference increased the overall median distance by approximately 1 mm—a modest and clinically manageable increment. In VR, the same transition produced a 2.5-fold larger median increase, with blind performance crossing the 2 mm clinical threshold. This asymmetry suggests that observers benefited more from AI-guided reference support in VR than in CIs, and that the two environments placed different demands on unaided spatial localisation. Critically, when AI guidance was present, VR median accuracy matched or exceeded that of CIs—a finding that motivates closer examination of the specific error sources that emerge without reference support.

Intraobserver reproducibility in CIs was consistent across all four participants, with medians remaining below 2.1 mm and all means below 3.4 mm, aligned with published benchmarks [32,33]. In VR, median values were similarly stable (2.07–2.96 mm) despite substantially higher means for two participants—a pattern consistent with sporadic outlier placements rather than systematic instability.

Interobserver reproducibility followed similar patterns: in CIs, medians remained stable across rounds with a modest increase attributable to the removal of the DL reference rather than observer inconsistency. In VR, mean interobserver distances were higher, but medians remained substantially lower and stable across blind rounds, indicating that observers developed consistent spatial strategies rather than performing randomly—consistent with findings that accuracy reaches a stable plateau across repeated VR sessions [46]. The condylar and gonion landmarks lie on broadly curved bony surfaces without discrete boundaries, requiring estimation across a gradual contour—a characteristic that challenges consistent localisation in both manual and automated approaches [32,33,35] and contributed to the elevated variability observed for these landmarks in both environments.

The landmarks at the inferior midline of the mandible presented a contrasting pattern in interobserver reproducibility. Gn showed consistently elevated interobserver variability across both environments and rounds, with Me following a similar pattern, particularly in VR. Unlike the condylar and gonion landmarks, this variability was not accompanied by the corresponding intraobserver instability—suggesting that observers located these points consistently within their own spatial framework, but that individual interpretations of the mandibular midline diverged systematically between observers. This pattern mirrors the well-documented interobserver inconsistency reported for Gn and Me in conventional 2D cephalometry [32,33], indicating that the underlying challenge is definitional rather than technical: both points lack a discrete anatomical boundary and are defined by functional criteria that admit multiple valid interpretations.

Observer accuracy and reproducibility were comparable between LD and uLD protocols in both environments, with overall median differences below 0.3 mm across all conditions. The uLD protocol thus appears viable for cephalometric workflows in both CIs and VR, consistent with the image-quality findings of the preceding pilot study [49]. This is relevant across all patient groups, as the ALARA principle mandates continuous dose optimisation [25,26] and repeated CBCT imaging is common in orthognathic and orthodontic contexts [53].

The NASA-TLX findings from the present study are consistent with the broader VR literature: immersive 3D visualisation has been shown to confer advantages in spatially demanding clinical tasks across medical specialties [54,55]. In Session 1, when DL coordinates were visible, observers reported lower mental demand and higher perceived performance than in subsequent blind sessions. The speed and freedom of 3D interaction were highlighted as genuine advantages, and perceived workload decreased progressively in VR—in contrast to CIs, where workload increased with blind landmarking—suggesting that clinical adoption of VR-based workflows is likely to be actively supported by specialist users. However, given the small number of observers, individual variation was substantial, and group-level workload patterns should be interpreted with caution.

As AI-based landmarking continues to improve in accuracy [35], its integration as a real-time assistive layer within VR—providing suggested coordinates that the radiologist can accept, adjust, or reject—represents a technically achievable next step. This human–AI collaborative model is likely to reduce the outlier placements that currently limit observer performance in VR and may bring immersive cephalometric workflows to clinical readiness. Expert feedback in VR has been shown to improve annotation quality and interobserver agreement for mandibular structures on CBCT models, with controller motion stabilisation explicitly identified as a critical technical requirement—a finding corroborated by the log-based analysis of controller interaction artefacts in the present study [56]. More broadly, interactive AI with expert input has demonstrated measurable improvements in medical image annotation tasks [57], supporting this direction.

The median-based reporting strategy adopted throughout was specifically chosen to limit the influence of outlier placements on the descriptive summary; the readiness requirements outlined below reflect this outlier-robust approach. The VR platform evaluated here remains a research tool; further software development is required before clinical evaluation would be appropriate. The performance gap is concentrated at a small number of anatomically demanding landmarks and attributable to specific software limitations rather than to immersive 3D visualisation itself.

Four targeted software developments are required for clinical readiness, in order of priority: first, automated real-time consistency checks between parallel coordinate logs (e.g., WorldSpace and LocalSpace), which would flag anomalous session-level discrepancies of the kind identified retrospectively in this study—a safeguard made more critical by the fact that a smaller-magnitude logging error of this type might not be noticed by the user at all; second, controller motion stabilisation and landmark locking mechanisms to reduce handheld input error; this reflects a fundamental characteristic of current VR input devices, where, in the absence of a confirmation lock, the recorded coordinate reflects controller position at the moment of button release, introducing physiological micromovement as an irreducible source of positional uncertainty that has no equivalent in mouse-based interaction; third, integration of cross-sectional depth reference within the VR interface—whether through MPR navigation or an equivalent approach; fourth, device-specific validation of VR display quality against DICOM calibration standards [29,41,42,49].

### 4.1. Limitations of the Study

This pilot design imposes several limitations. The small sample size and single-platform setting restrict generalisability. A further limitation concerns the asymmetry in cross-sectional information available between environments: CI provided full multiplanar reconstruction (MPR) alongside the 3D surface model, whereas VR landmarking was restricted to the surface-rendered model alone. This is a confound rather than a pure test of visualisation modality, as the two environments differed not only in how spatial information was presented but in what information was available. Landmarks with inherent depth ambiguity—most clearly PNS in this dataset, but potentially others—are disproportionately affected by this asymmetry.

Seven VR placements were absent from the coordinate logs due to a software recording failure; manual inspection confirmed these placements were performed, but the absence of stored coordinates introduced minor incompleteness in the VR dataset.

A direct numerical comparison with other radiological VR applications is not feasible given the task-specific nature of cephalometric landmarking; however, the controller precision limitations and display calibration challenges identified here are consistent with those reported across radiological VR contexts [29,41,42], suggesting that these represent platform-level constraints rather than task-specific failures. Studies in surgical planning and interventional radiology similarly emphasise the need for device-specific validation of VR display quality to ensure diagnostic reliability [29,41,42]. These parallels suggest that the limitations observed reflect broader technical constraints of current VR platforms in radiology rather than characteristics unique to this application or its observers.

Furthermore, the descriptive analytical approach—necessitated by the pilot sample size—did not permit formal reliability quantification (e.g., intraclass correlation coefficients, Bland–Altman limits of agreement); future studies with larger, pre-registered samples should incorporate these to establish accuracy and reproducibility estimates with defined precision.

### 4.2. Recommendations for Future Research

These limitations directly inform future directions: larger and more diverse observer cohorts, multi-platform comparisons, and purpose-built VR software with MPR navigation and PACS connectivity are immediate priorities. Prospective orthodontic studies would provide confirmatory data on uLD protocol feasibility across repeated imaging. The integration of DL automated landmarking with VR-based expert verification—building on emerging evidence that interactive AI in VR improves annotation quality and reduces interobserver variation [56,57]—warrants prioritisation in future research.

## 5. Conclusions

The accuracy and reproducibility of 3D cephalometric landmarking are influenced by both the imaging protocol and the visualisation environment employed. This pilot study demonstrates that CIs met established clinical accuracy and reproducibility benchmarks, confirming their suitability as a reference standard for CMF CASS, and that radiation dose reduction to uLD levels had no meaningful effect on landmarking performance in either environment at the precision achievable with this pilot sample—a preliminary indication of uLD feasibility for cephalometric landmarking in both orthognathic surgical planning and orthodontic assessment, worth confirming in larger, multi-platform studies. In VR, median accuracy was comparable to CIs when AI guidance was available, and observer acceptance was consistently positive. Without AI guidance, reproducibility was lower and outlier rates higher than in CIs, attributable to controller interaction limitations and surface rendering constraints rather than immersive visualisation itself. The landmark-level error patterns reflect challenges well documented in 3D cephalometry, suggesting that fundamental anatomical difficulties persist across visualisation environments and are not resolved by immersive technology alone. The foundations for VR-based cephalometric workflows are in place, but targeted software developments are required before clinical implementation alongside CIs, at which point comparable dose-level evaluation in VR would also be warranted. Integration of AI-assisted landmarking within VR represents the most promising direction for this development.

## Figures and Tables

**Table 1:** Summary of CBCT exposure parameters by protocol (*n* = 10 patients per protocol).

Protocol	*n*	kVp	mA	Exposure (s)	FOV (cm)	DAP (mGy·cm^2^)	Voxel (mm)
LD	10	90	14	4.50	16 × 13–20 × 15	722.1–758.0 [749.4]	0.3
uLD	10	120	3–5	2.50–3.20	14 × 11–18 × 17	218.1–414.5 [304.0]	0.3

DAP = dose–area product (mGy·cm^2^); values in brackets represent mean DAP. FOV range reflects variation across the 20 patients. LD = low-dose; uLD = ultra-low-dose.

**Table 2:** Round 1 accuracy: 3D Euclidean distances from DL reference coordinates (mm). Part A shows observer-level summary; Part B shows landmark-level detail.

	CI		VR	
	Mean (mm)	Median (mm)	Mean (mm)	Median (mm)
**Part A: Observer-level**				
E1	1.21	1.41	1.09	0.98
E2	0.56	0.54	2.44	0.80
E3	1.18	1.41	2.97	0.78
E4	1.26	1.41	1.63	1.54
**Overall**	**1.05**	**1.00**	**2.03**	**0.97**
**Part B: Landmark-level**				
N	1.03	1.00	1.25	1.09
CoR	1.10	1.06	3.14	1.02
CoL	1.07	1.02	4.01	0.94
PNS	1.08	1.01	1.24	1.04
ANS	1.04	1.00	4.70	1.30
A-point	0.97	1.00	0.95	0.87
Gn	0.97	1.00	0.94	0.87
Me	1.05	1.00	1.00	0.90
GoR	1.01	1.00	1.15	0.99
GoL	1.16	1.41	1.94	0.91
**Overall**	**1.05**	**1.00**	**2.03**	**0.97**

CI = computer interface; VR = virtual reality. DL = deep learning. Values represent descriptive statistics (mean, median) only; no inferential statistical testing was performed, consistent with the descriptive analytical approach adopted throughout (Section 2.9).

**Table 3:** Deviation from DL reference coordinates during blind landmarking (Rounds 2 and 3) by landmark and environment (mm).

	CI				VR			
Landmark	R2 Mean	R2 Median	R3 Mean	R3 Median	R2 Mean	R2 Median	R3 Mean	R3 Median
N	2.15	1.87	2.05	1.73	3.50	2.90	3.29	2.80
CoR	2.14	1.41	4.03	1.87	6.30	3.82	6.20	3.82
CoL	2.38	2.24	2.23	2.24	7.10	3.77	7.90	3.49
PNS	1.93	2.00	1.68	1.41	15.60	3.24	7.72	3.04
ANS	1.67	1.44	1.73	1.57	5.86	2.77	5.97	2.85
A-point	2.23	1.73	2.29	2.12	3.56	2.43	3.75	3.11
Gn	2.87	2.24	2.92	2.64	6.08	4.95	6.06	4.93
Me	2.62	2.45	2.52	2.45	4.41	4.04	4.23	3.54
GoR	3.02	2.83	2.86	2.45	5.02	4.13	5.01	4.64
GoL	2.46	2.24	3.93	2.00	6.25	4.69	8.36	5.39
**Overall**	**2.35**	**2.03**	**2.62**	**2.00**	**6.37**	**3.58**	**5.85**	**3.68**

CI = computer interface; VR = virtual reality. R2 = Round 2; R3 = Round 3. DL = deep learning. Values represent descriptive statistics (mean, median) only; no inferential statistical testing was performed, consistent with the descriptive analytical approach adopted throughout (Section 2.9).

**Table 4:** Landmark placement completeness and outlier summary by condition and round.

Condition	Round	Total Possible Placements	Completed *n* (%)	Outliers > 10 mm	Outlier Rate (%)	Error Types Identified
CI	R1	800	763 (95.4%) *	1	0.13	Isolated misplacement (*n* = 1)
	R2	800	740 (92.5%) *	—	—	
	R3	800	700 (87.5%) *	—	—	
VR	R1	800	793 (99.1%) †	22	2.77	Controller slips (*n* = 4); software logging error (*n* = 18) ‡
	R2	800	800 (100%)	—	—	
	R3	800	800 (100%)	—	—	
Combined	R1	1600	1556 (97.3%)	23	1.48	

* CI completeness: placements missing due to software logging and file export issues; not attributable to evaluator omission. † VR completeness R1: 7 placements missing due to software logging failure (LocalSpace coordinates confirmed present). ‡ ANS systematically displaced ~29 mm in one session (18/19 patients) due to a session-level software logging error; excluded from accuracy analysis. Controller slips: 4 placements with anatomically impossible displacements (138–300 mm). — = not assessed. CI = computer interface; VR = virtual reality. R1 = Round 1; R2 = Round 2; R3 = Round 3. Outlier threshold: >10 mm 3D Euclidean distance from DL reference.

**Table 5:** Intraobserver reproducibility (Round 2 vs. Round 3): 3D Euclidean distances (mm). Part A shows observer-level summary; Part B shows landmark-level detail.

	CI		VR	
	Mean (mm)	Median (mm)	Mean (mm)	Median (mm)
**Part A: Observer-level**				
E1	1.98	1.73	3.88	2.07
E2	3.36	1.99	2.83	2.62
E3	2.37	2.05	6.29	2.96
E4	2.98	1.98	7.04	2.96
**Part B: Landmark-level**				
N	1.96	1.73	2.40	2.09
CoR	4.31	2.24	3.28	3.20
CoL	2.68	2.24	3.83	2.66
PNS	1.61	1.41	19.37 *	2.50
ANS	1.75	1.33	2.38	2.21
A-point	2.11	1.66	3.07	2.39
Gn	2.15	2.00	3.35	3.10
Me	2.08	2.00	3.33	3.13
GoR	3.07	2.45	3.55	2.78
GoL	4.55	2.24	5.49	2.95
**Overall**	**2.63**	**2.00**	**5.01**	**2.66**

* PNS VR mean driven by outlier placements (median: 2.50 mm). CI = computer interface; VR = virtual reality. Values represent descriptive statistics (mean, median) only; no inferential statistical testing was performed, consistent with the descriptive analytical approach adopted throughout (Section 2.9).

**Table 6:** Interobserver reproducibility (Rounds 2 and 3): 3D Euclidean distances (mm). Part A shows observer-pair distances; Part B shows landmark-level detail.

	CI				VR			
	R2 Mean	R2 Median	R3 Mean	R3 Median	R2 Mean	R2 Median	R3 Mean	R3 Median
**Part A: Observer pairs**								
E1–E2	2.75	2.34	3.44	2.11	5.82	3.35	5.18	3.88
E1–E3	2.71	1.87	3.17	1.83	8.93	3.85	6.80	4.55
E1–E4	2.49	2.16	3.57	2.45	7.79	4.39	6.88	4.01
E2–E3	2.99	2.59	3.43	2.12	7.45	4.15	6.17	4.25
E2–E4	2.87	2.45	3.52	2.24	5.69	3.46	5.71	2.80
E3–E4	2.30	1.98	3.22	2.11	9.35	4.25	7.81	3.87
**Part B: Landmark-level**								
N	2.64	2.24	2.36	2.00	4.13	3.67	3.67	3.20
CoR	2.38	2.24	6.34	2.00	4.12	3.65	4.26	4.19
CoL	2.80	2.24	2.20	2.24	3.95	3.61	6.03	3.83
PNS	1.93	1.73	1.97	1.73	28.67 †	3.84	12.95 †	3.86
ANS	1.74	1.41	1.87	1.41	3.15	2.98	2.74	2.38
A-point	2.34	2.00	2.64	2.24	3.91	2.85	3.36	3.19
Gn	3.45	3.46	3.73	3.61	8.28	7.06	8.35	7.46
Me	3.35	3.16	3.52	3.32	6.01	5.37	5.70	5.25
GoR	3.01	2.83	3.92	3.46	6.61	5.25	6.58	6.31
GoL	3.23	2.83	5.36	2.83	6.24	5.79	10.61	7.05
**Overall**	**2.69**	**2.24**	**3.39**	**2.24**	**7.51**	**4.16**	**6.42**	**4.20**

† PNS VR means driven by outlier placements (medians: 3.84–3.86 mm). CI = computer interface; VR = virtual reality. R2 = Round 2; R3 = Round 3. Values represent descriptive statistics (mean, median) only; no inferential statistical testing was performed, consistent with the descriptive analytical approach adopted throughout (Section 2.9).

**Table 7:** Dose-stratified accuracy and intraobserver reproducibility by environment.

	CI		VR	
Metric	uLD	LD	uLD	LD
**Round 1 accuracy vs. DL reference**				
Mean (mm)	1.05	1.04	1.79	2.27
Median (mm)	1.00	1.00	0.98	0.96
**Intraobserver reproducibility (Round 2 vs. Round 3)**				
Mean (mm)	2.66	2.60	5.15	4.86
Median (mm)	1.73	2.00	2.78	2.56

LD = low-dose (90 kVp, 14 mA); uLD = ultra-low-dose (120 kVp, 3–5 mA); DL = deep learning. Values represent grand mean/median across all landmarks and observers; all are descriptive statistics, with no inferential testing performed, consistent with the descriptive analytical approach adopted throughout (Section 2.9).

## Data Availability

The datasets used for image quality analysis were provided by the Wellbeing Services County of Pirkanmaa (Tampere University Hospital). They are not publicly available, and their use is restricted under Finnish law and the EU General Data Protection Regulation (GDPR).

## Supplementary Materials

The following supporting information can be downloaded at: https://www.mdpi.com/article/10.3390/diagnostics16162493/s1, Table S1: Landmark placement completeness and outlier frequencies by condition and anatomical landmark (Round 1); Table S2: Number of landmark placements deviating >10 mm between intraobserver rounds (Round 2 vs. Round 3), by observer and landmark.

## Abbreviations

The following abbreviations are used in this manuscript:

ALARA	as low as reasonably achievable
ANS A-point	anterior nasal spine subspinale
CASS	computer-aided surgical simulation
CBCT	cone-beam computed tomography
CI	computer interface
CMF	craniomaxillofacial
CoL	condylion left
CoR	condylion right
DAP	dose–area product
DICOM	digital imaging and communications in medicine
DL	deep learning
FOV	field of view
Gn	gnathion
GoL	gonion left
GoR	gonion right
LD	low-dose
Me	menton
MPR	multiplanar reconstruction
N	nasion
NASA-TLX	NASA task load index
PACS	picture archiving and communication system
PNS	posterior nasal spine
uLD	ultra-low-dose
VR	virtual reality

## References

[1] Smith-Bindman R., Miglioretti D.L., Johnson E., Lee C., Feigelson H.S., Flynn M., Greenlee R.T., Kruger R.L., Hornbrook M.C., Roblin D. (2012). Use of Diagnostic Imaging Studies and Associated Radiation Exposure for Patients Enrolled in Large Integrated Health Care Systems, 1996-2010. JAMA.

[2] Sakas G. (2002). Trends in Medical Imaging: From 2D to 3D. Comput. Graph..

[3] Wrzosek M., Peacock Z., Laviv A., Goldwaser B., Ortiz R., Resnick C., Troulis M., Kaban L. (2016). Comparison of Time Required for Traditional versus Virtual Orthognathic Surgery Treatment Planning. Int. J. Oral Maxillofac. Surg..

[4] Alkhayer A., Piffkó J., Lippold C., Segatto E. (2020). Accuracy of Virtual Planning in Orthognathic Surgery: A Systematic Review. Head Face Med..

[5] Hsu S.S.-P., Gateno J., Bell R.B., Hirsch D.L., Markiewicz M.R., Teichgraeber J.F., Zhou X., Xia J.J. (2013). Accuracy of a Computer-Aided Surgical Simulation Protocol for Orthognathic Surgery: A Prospective Multicenter Study. J. Oral Maxillofac. Surg..

[6] Xia J.J., Gateno J., Teichgraeber J.F., Christensen A.M., Lasky R.E., Lemoine J.J., Liebschner M.A. (2007). Accuracy of the Computer-Aided Surgical Simulation (CASS) System in the Treatment of Patients with Complex Craniomaxillofacial Deformity: A Pilot Study. J. Oral Maxillofac. Surg..

[7] Xia J.J., Shevchenko L., Gateno J., Teichgraeber J.F., Taylor T.D., Lasky R.E., English J.D., Kau C.H., McGrory K.R. (2011). Outcome Study of Computer-Aided Surgical Simulation in the Treatment of Patients with Craniomaxillofacial Deformities. J. Oral Maxillofac. Surg..

[8] Powcharoen W., Yang W., Li K.Y., Zhu W., Su Y. (2019). Computer-Assisted versus Conventional Freehand Mandibular Reconstruction with Fibula Free Flap: A Systematic Review and Meta-Analysis. Plast. Reconstr. Surg..

[9] Bartier S., Mazzaschi O., Benichou L., Sauvaget E. (2021). Computer-Assisted versus Traditional Technique in Fibular Free-Flap Mandibular Reconstruction: A CT Symmetry Study. Eur. Ann. Otorhinolaryngol. Head Neck Dis..

[10] Palla B., Callahan N. (2021). Does the Use of Computer-Assisted Surgery Affect the Margin Status in Resections of Ameloblastoma?. J. Oral Maxillofac. Surg..

[11] Bergeron L., Bonapace-Potvin M., Bergeron F. (2021). In-House 3D Model Printing for Acute Cranio-Maxillo-Facial Trauma Surgery: Process, Time, and Costs. Plast. Reconstr. Surg.—Glob. Open.

[12] Schwartz H. (2014). Does Computer-Aided Surgical Simulation Improve Efficiency in Bimaxillary Orthognathic Surgery?. Int. J. Oral Maxillofac. Surg..

[13] Ferri J., Druelle C., Schlund M., Bricout N., Nicot R. (2019). Complications in Orthognathic Surgery: A Retrospective Study of 5025 Cases. Int. Orthod..

[14] Bowe D., Gruber E., McLeod N. (2016). Nerve Injury Associated with Orthognathic Surgery. Part 1: UK Practice and Motor Nerve Injuries. Br. J. Oral Maxillofac. Surg..

[15] McLeod N., Bowe D. (2016). Nerve Injury Associated with Orthognathic Surgery. Part 2: Inferior Alveolar Nerve. Br. J. Oral Maxillofac. Surg..

[16] McLeod N., Bowe D. (2016). Nerve Injury Associated with Orthognathic Surgery. Part 3: Lingual, Infraorbital, and Optic Nerves. Br. J. Oral Maxillofac. Surg..

[17] Panula K., Finne K., Oikarinen K. (2001). Incidence of Complications and Problems Related to Orthognathic Surgery: A Review of 655 Patients. J. Oral Maxillofac. Surg..

[18] de Santana Santos T., Albuquerque K.M., Santos M.E.S.M., Laureano Filho J.R. (2012). Survey on Complications of Orthognathic Surgery among Oral and Maxillofacial Surgeons. J. Craniofac. Surg..

[19] Mendes D., Caputo F.M., Giachetti A., Ferreira A., Jorge J. (2019). A Survey on 3D Virtual Object Manipulation: From the Desktop to Immersive Virtual Environments.

[20] Kusnoto B. (2007). Two-Dimensional Cephalometry and Computerized Orthognathic Surgical Treatment Planning. Clin. Plast. Surg..

[21] Ayoub A., Pulijala Y. (2019). The Application of Virtual Reality and Augmented Reality in Oral & Maxillofacial Surgery. BMC Oral Health.

[22] Reyneke J.P., Ferretti C. (2021). Diagnosis and Planning in Orthognathic Surgery. Oral and Maxillofacial Surgery for the Clinician.

[23] Dot G., Rafflenbeul F., Arbotto M., Gajny L., Rouch P., Schouman T. (2020). Accuracy and Reliability of Automatic Three-Dimensional Cephalometric Landmarking. Int. J. Oral Maxillofac. Surg..

[24] Pauwels R. (2015). Cone Beam CT for Dental and Maxillofacial Imaging: Dose Matters. Radiat. Prot. Dosim..

[25] Tamminen P., Järnstedt J., Numminen J., Lehtinen A., Lehtimäki L., Rautiainen M., Kivekäs I. (2023). Ultra-Low-Dose CBCT: New Cornerstone of Paranasal Sinus Imaging. Rhinology.

[26] Tamminen P., Järnstedt J., Lehtinen A., Numminen J., Lehtimäki L., Rautiainen M., Kivekäs I. (2023). Ultra-Low-Dose CBCT Scan: Rational Map for Ear Surgery. Eur. Arch. Oto-Rhino-Laryngol..

[27] Dos Santos R.M.G., De Martino J.M., Neto F.H., Passeri L.A. (2018). Cone-Beam Computed Tomography-Based Three-Dimensional McNamara Cephalometric Analysis. J. Craniofac. Surg..

[28] Gateno J., Xia J.J., Teichgraeber J.F. (2011). New 3-Dimensional Cephalometric Analysis for Orthognathic Surgery. J. Oral Maxillofac. Surg..

[29] Javvaji C.K., Reddy H., Vagha J.D., Taksande A., Kommareddy A., Reddy N.S., Sudheesh Reddy N. (2024). Immersive Innovations: Exploring the Diverse Applications of Virtual Reality (VR) in Healthcare. Cureus.

[30] Deng H.H., Liu Q., Chen A., Kuang T., Yuan P., Gateno J., Kim D., Barber J.C., Xiong K.G., Yu P. (2023). Clinical Feasibility of Deep Learning-Based Automatic Head CBCT Image Segmentation and Landmark Detection in Computer-Aided Surgical Simulation for Orthognathic Surgery. Int. J. Oral Maxillofac. Surg..

[31] Gateno J., Xia J.J., Teichgraeber J.F., Christensen A.M., Lemoine J.J., Liebschner M.A., Gliddon M.J., Briggs M.E. (2007). Clinical Feasibility of Computer-Aided Surgical Simulation (CASS) in the Treatment of Complex Cranio-Maxillofacial Deformities. J. Oral Maxillofac. Surg..

[32] de Oliveira A.E.F., Cevidanes L.H.S., Phillips C., Motta A., Burke B., Tyndall D. (2009). Observer Reliability of Three-Dimensional Cephalometric Landmark Identification on Cone-Beam Computerized Tomography. Oral Surg. Oral Med. Oral Pathol. Oral Radiol. Endodontology.

[33] Damstra J., Fourie Z., Slater J.J.H., Ren Y. (2011). Reliability and the Smallest Detectable Difference of Measurements on 3-Dimensional Cone-Beam Computed Tomography Images. Am. J. Orthod. Dentofac. Orthop..

[34] Serafin M., Baldini B., Cabitza F., Carrafiello G., Baselli G., Del Fabbro M., Sforza C., Caprioglio A., Tartaglia G.M. (2023). Accuracy of Automated 3D Cephalometric Landmarks by Deep Learning Algorithms: Systematic Review and Meta-Analysis. La Radiol. Medica.

[35] Sahlsten J., Järnstedt J., Jaskari J., Naukkarinen H., Mahasantipiya P., Charuakkra A., Vasankari K., Hietanen A., Sundqvist O., Lehtinen A. (2024). Deep Learning for 3D Cephalometric Landmarking with Heterogeneous Multi-Center CBCT Dataset. PLoS ONE.

[36] Liu B., Liu C., Xiong Y., Zhu H., Zeng W., Chen J., Guo J., Liu W., Tang W. (2025). Accuracy and Reliability of 3D Cephalometric Landmark Detection with Deep Learning. Eur. J. Med. Res..

[37] Kin T., Nakatomi H., Shono N., Nomura S., Saito T., Oyama H., Saito N. (2017). Neurosurgical Virtual Reality Simulation for Brain Tumor Using High-Definition Computer Graphics: A Review of the Literature. Neurol. Med.-Chir..

[38] Bairamian D., Liu S., Eftekhar B. (2019). Virtual Reality Angiogram vs 3-Dimensional Printed Angiogram as an Educational Tool—A Comparative Study. Neurosurgery.

[39] Javaid M., Haleem A., Singh R.P., Khan S. (2022). Understanding Roles of Virtual Reality in Radiology. Internet Things Cyber-Phys. Syst..

[40] Rantamaa H.-R., Kangas J., Jordan M., Mehtonen H., Mäkelä J., Ronkainen K., Turunen M., Sundqvist O., Syrjä I., Järnstedt J. (2022). Evaluation of Voice Commands for Mode Change in Virtual Reality Implant Planning Procedure. Int. J. Comput. Assist. Radiol. Surg..

[41] Tortora M., Luppi A., Pacchiano F., Marisei M., Grassi F., Werner H., Kitamura F.C., Tortora F., Caranci F., Ferraciolli S.F. (2025). Current Applications and Future Perspectives of Extended Reality in Radiology. La Radiol. Medica.

[42] Stucki J., Dastgir R., Baur D.A., Quereshy F.A. (2024). The Use of Virtual Reality and Augmented Reality in Oral and Maxillofacial Surgery: A Narrative Review. Oral Surg. Oral Med. Oral Pathol. Oral Radiol..

[43] Lee S.M., Coimbra Baffi H., Ola T., Tsang B., Gupta A., Faingold R., Stimec J., Doria A.S. (2025). Clinical Applications of Virtual and Augmented Reality in Radiology: A Scoping Review. J. Clin. Med..

[44] Moro C., Birt J., Stromberga Z., Phelps C., Clark J., Glasziou P., Scott A.M. (2021). Virtual and Augmented Reality Enhancements to Medical and Science Student Physiology and Anatomy Test Performance: A Systematic Review and Meta-analysis. Anat. Sci. Educ..

[45] Nalampang S., Yanchinda J., Prapayasatok S., Charuakkra A., Kaewkhong K., Järnstedt J. (2026). Digital and Immersive Approaches to Anatomy Education: A Pilot Comparative Study of CI, VR, and Hybrid Learning in Implant Planning. BMC Med. Educ..

[46] Jo Y.-J., Choi J.-S., Kim J., Kim H.-J., Moon S.-Y. (2021). Virtual Reality (VR) Simulation and Augmented Reality (AR) Navigation in Orthognathic Surgery: A Case Report. Appl. Sci..

[47] Lo L.-J., Lin H.-H. (2023). Applications of Three-Dimensional Imaging Techniques in Craniomaxillofacial Surgery: A Literature Review. Biomed. J..

[48] Koivisto J., Wolff J., Pauwels R., Kaasalainen T., Suomalainen A., Stoor P., Horelli J., Suojanen J. (2024). Assessment of Cone-Beam CT Technical Image Quality Indicators and Radiation Dose for Optimal STL Model Used in Visual Surgical Planning. Dentomaxillofacial Radiol..

[49] Järnstedt J., Mehtonen H., Kangas J., Ronkainen K., Mäkelä J., Nalampang S., Mahasantipiya P., Charuakkra A., Panyarak W., Naderi A. (2026). Influence of Cone Beam Computed Tomography Radiation Dose on Image Quality and Usability in Virtual Reality and Traditional Computer Interfaces. Appl. Sci..

[50] Mehtonen H., Järnstedt J., Kangas J., Kumar S., Rinta-Kiikka I., Raisamo R. (2023). Evaluation of Properties and Usability of Virtual Reality Interaction Techniques in Craniomaxillofacial Computer-Assisted Surgical Simulation. Proceedings of the 35th Australian Computer-Human Interaction Conference (OzCHI 2023), Wellington, New Zealand, 2–6 December 2023.

[51] Hart S.G., Staveland L.E. (1988). Development of NASA-TLX (Task Load Index): Results of Empirical and Theoretical Research. Advances in Psychology.

[52] Suhail S., Harris K., Sinha G., Schmidt M., Durgekar S., Mehta S., Upadhyay M. (2022). Learning Cephalometric Landmarks for Diagnostic Features Using Regression Trees. Bioengineering.

[53] Polizzi A., Serra S., Leonardi R. (2024). Use of CBCT in Orthodontics: A Scoping Review. J. Clin. Med..

[54] Javaid M., Haleem A. (2020). Virtual Reality Applications toward Medical Field. Clin. Epidemiol. Glob. Health.

[55] Gómez-Tone H.C., Martin-Gutierrez J., Bustamante-Escapa J., Bustamante-Escapa P. (2021). Spatial Skills and Perceptions of Space: Representing 2D Drawings as 3D Drawings inside Immersive Virtual Reality. Appl. Sci..

[56] Orsmaa L., Saukkoriipi M., Kangas J., Rasouli N., Järnstedt J., Mehtonen H., Sahlsten J., Jaskari J., Kaski K., Raisamo R. (2026). Interactive AI Annotation of Medical Images in a Virtual Reality Environment. Int. J. Comput. Assist. Radiol. Surg..

[57] Saukkoriipi M., Sahlsten J., Jaskari J., Orsmaa L., Kangas J., Rasouli N., Raisamo R., Hirvonen J., Mehtonen H., Järnstedt J. (2025). Interactive 3D Segmentation for Primary Gross Tumor Volume in Oropharyngeal Cancer. Sci. Rep..

