# Supplementary material for: Immersive Virtual Reality for 3D Cephalometric Landmarking Across Low-Dose and Ultra-Low-Dose CBCT: A Pilot Comparison with a Conventional Computer Interface
Source: Diagnostics (Basel). 2026 Aug 7;16(16):2493. doi: 10.3390/diagnostics16162493 (PMC13511459; doi:10.3390/diagnostics16162493)
Supplement: Supplementary file 1 [file diagnostics-16-02493-s001.zip › SUPPLEMENTARY.pdf]

**SUPPLEMENTARY**

# Immersive Virtual Reality for 3D Cephalometric Landmarking Across Low-Dose and Ultra-Low-Dose CBCT: A Pilot Comparison with Conventional Computer Interface

**Jorma Järnstedt [1,2,*,†], Helena Mehtonen [1,2,3,†], Jari Kangas [3], Hanna Naukkarinen [4], Kimmo Ronkainen [3], John Mäkelä [3], Sakarat Nalampang [5], Phattaranant Mahasantipiya [5], Arnon Charuakkra [5], Wannakamon Panyarak [5], Irina Rinta-Kiikka [1,2] and Roope Raisamo [3,*]**

[1] Department of Radiology, Tampere University Hospital, Wellbeing Services County of Pirkanmaa,
33250 Tampere, Finland
[2] Faculty of Medicine and Health Technology, Tampere University, 33014 Tampere, Finland
[3] TAUCHI Research Center, Faculty of Information Technology and Communication Sciences,
Tampere University, 33100 Tampere, Finland
[4] Faculty of Medicine, University of Helsinki
[5] Division of Oral and Maxillofacial Radiology, Department of Oral Biology and Diagnostic Sciences, Faculty of Dentistry, Chiang Mai University, Chiang Mai 50200, Thailand

* Correspondence: jorma.jarnstedt@pirha.fi (J.J.); roope.raisamo@tuni.fi (R.R.)
† Jorma Jarnstedt and Helena Mehtonen are co-first authors of this manuscript, having contributed equally to the study.

**Supplementary Table S1.**

Landmark placement completeness and outlier frequencies by condition and anatomical landmark, Round 1.

| Landmark | Total placements | Completed n (%) | Outliers >10 mm | Outlier rate (%) | Error type |
|---|---|---|---|---|---|
| **CI (conventional screen)** | | | | | |
| N | 80 | 80 (100%) | 0 | 0.00 | — |
| CoR | 80 | 80 (100%) | 0 | 0.00 | — |
| CoL | 80 | 80 (100%) | 0 | 0.00 | — |
| PNS | 80 | 80 (100%) | 0 | 0.00 | — |
| ANS | 80 | 80 (100%) | 0 | 0.00 | — |
| A-point | 80 | 80 (100%) | 0 | 0.00 | — |
| Gn | 80 | 80 (100%) | 1 | 1.25 | Isolated misplacement |
| Me | 80 | 80 (100%) | 0 | 0.00 | — |
| GoR | 80 | 80 (100%) | 0 | 0.00 | — |
| GoL | 77 | 77 (96.3%) * | 0 | 0.00 | — |
| **CI Total** | **800** | **763 (95.4%)** | **1** | **0.13** | |
| **VR (virtual reality)** | | | | | |
| N | 77 | 77 (96.3%) † | 0 | 0.00 | — |
| CoR | 80 | 79 (98.8%) | 1 | 1.27 | Controller slip (~300 mm) |
| CoL | 80 | 80 (100%) | 2 | 2.50 | Controller slips (~300 mm, ~139 mm) |
| PNS | 80 | 80 (100%) | 0 | 0.00 | — |
| ANS | 77 | 76 (98.7%) † | 18 | 23.68 | Software logging error (~29 mm, 1 session) |
| A-point | 80 | 80 (100%) | 0 | 0.00 | — |
| Gn | 80 | 80 (100%) | 0 | 0.00 | — |
| Me | 80 | 80 (100%) | 0 | 0.00 | — |
| GoR | 80 | 80 (100%) | 0 | 0.00 | — |
| GoL | 80 | 80 (100%) | 1 | 1.25 | Controller slip (~137 mm) |
| **VR Total** | **800** | **793 (99.1%)** | **22** | **2.77** | |
| **Combined Total** | **1600** | **1556 (97.3%)** | **23** | **1.48** | |

*Red shading = cells with ≥1 outlier placement. — = no outliers. * CI Gonion Left: n = 77 (file export issue; not attributable to evaluator omission). † VR Nasion and ANS: reduced n due to software logging failure (LocalSpace coordinates confirmed present; not attributable to evaluator omission). CI = computer interface; VR = virtual reality. Outlier threshold: >10 mm 3D Euclidean distance from DL reference.*

**Supplementary Table S2.** Number of landmark placements deviating >10 mm between intraobserver rounds (Round 2 vs. Round 3), by observer and landmark.

**Part A: CI — Placements >10 mm between intraobserver rounds (Round 2 vs. Round 3)**

| Landmark | E1 | E2 | E3 | E4 | Total |
|---|---|---|---|---|---|
| N | — | — | — | — | — |
| CoR | — | — | — | — | — |
| CoL | — | 1 | 1 | — | 2 |
| PNS | 1 | — | 1 | 2 | 4 |
| ANS | — | — | — | — | — |
| A-point | — | 1 | 2 | — | 3 |
| Gn | — | — | — | — | — |
| Me | — | — | — | — | — |
| GoR | — | — | 1 | — | 1 |
| GoL | — | — | 4 | 1 | 5 |
| **Total** | **1** | **2** | **10** | **3** | **16** |

**Part B: VR — Placements >10 mm between intraobserver rounds (Round 2 vs. Round 3)**

| Landmark | E1 | E2 | E3 | E4 | Total |
|---|---|---|---|---|---|
| N | — | — | — | — | — |
| CoR | — | — | — | — | — |
| CoL | — | 1 | 1 | — | 2 |
| PNS | 1 | — | 2 | — | 3 |
| ANS | — | — | — | — | — |
| A-point | — | 1 | 2 | — | 3 |
| Gn | — | — | — | — | — |
| Me | — | — | — | — | — |
| GoR | — | — | — | — | — |
| GoL | — | — | 4 | — | 4 |
| **Total** | **1** | **2** | **9** | **—** | **12** |

*Red shading = cells with ≥1 outlier placement. — = zero occurrences. E1–E4 = four CMF radiologists. CI = computer interface; VR = virtual reality.*
